# Reply to Pandey: IgG3 allotypes, modulation of antigen binding by constant domain changes, and therapeutic applications of Fc engineering

**DOI:** 10.1073/pnas.2306562120

**Published:** 2023-05-22

**Authors:** Arman Izadi, Wael Bahnan, Mats Ohlin, Pontus Nordenfelt

**Affiliations:** ^a^Department of Clinical Sciences Lund, Infection Medicine, Faculty of Medicine, Lund University, 221 84, Lund, Sweden; ^b^Department of Immunotechnology, Lund University, 221 00 Lund, Sweden; ^c^SciLifeLab Drug Discovery and Development, Lund University, 221 00 Lund, Sweden; ^d^Department of Laboratory Medicine, Clinical Microbiology, Skåne University Hospital Lund, Lund University, 221 85 Lund, Sweden

It was with great interest that we read the comments made by Pandey (1) on our recent study (2), showing that subclass-switched IgG3 mAbs display more potent complement activation, complement-mediated phagocytosis, and Fc-mediated phagocytosis compared to their IgG1 counterparts. In addition, we demonstrated that altering the constant domain modulates the antigen-binding properties of the mAbs.

We fully agree with Pandey’s opinion that the influence of the constant region on binding, fine specificity, and idiotype expression has been described in the past (3, 4) but has been largely overlooked. The constant domain has a role not only for mediating effector functions such as complement activation, antibody-dependent cellular cytotoxicity, and phagocytosis, but also for antigen-binding properties per se. This is very important to consider when evaluating mAb functionality.

As Pandey pointed out, there is substantial sequence diversity in immunoglobulin heavy chain constant genes, particularly for IgG3, some of which translate into differences in the protein sequence. The recognition of allotypes (5) was evidence of this fact. Indeed, diversity in the immunoglobulin heavy chain constant genes may be larger than previously thought (6). The residue 435 mentioned by Pandey is directly involved in the binding of antibodies to FcRn. This codon is associated with a single-nucleotide polymorphism (SNP rs4042056) that defines the arginine/histidine difference between different IgG3 allotypes and thereby affects the half-life of the encoded protein (7), where the shorter half-life arginine variant is the most common.

We certainly agree that future evaluation of different IgG3 variants offers opportunities to identify better candidates for therapeutic applications dependent on effector functions or antibody half-life. Fc engineering using this information could fine-tune complement activation capacity, FcR affinity, or FcRn affinity for improved half-life. Indeed, such an approach was taken to enhance serum half-life and reduce the effector functions of a therapeutic antibody cocktail (8) intended for prophylactic treatment of SARS-CoV-2 infection. In addition, IgG3 mAbs, when produced at a large scale, experience aggregation issues. However, by Fc engineering of N392K and M397V, also this issue has been shown to be addressable (9). Mounting evidence shows that IgG3 mAbs have potent in vitro function for different antigens and pathogens (2, 7, 10, 11), combined with the ability to address its limitations in half-life and aggregation by Fc engineering, which motivates its applicability as a therapeutic backbone for mAbs. We very much look forward to the evolution of this field in relation to antibodies specific for SARS-CoV-2 and beyond in the years to come.
